# Supplementary material for: Risk scores, label bias, and everything but the kitchen sink
Source: Sci Adv. 2024 Mar 29;10(13):eadi8411. doi: 10.1126/sciadv.adi8411 (PMC10980258; doi:10.1126/sciadv.adi8411)
Supplement: 20240329-1 [file sciadv.adi8411.v1.pdf]

COMPUTER SCIENCE

Risk scores, label bias, everything but the kitchen sink

Michael Zanger-Tishler[1]*, Julian Nyarko[2], Sharad Goel[3]

Copyright © 2024 The Authors, some rights reserved; exclusive licensee American Association for the Advancement of Science. No claim to original U.S. Government Works. Distributed under a Creative Commons Attribution License 4.0 (CC BY).

In designing risk assessment algorithms, many scholars promote a "kitchen sink" approach, reasoning that more information yields more accurate predictions. We show, however, that this rationale often fails when algorithms are trained to predict a proxy of the true outcome, for instance, predicting arrest as a proxy for criminal behavior. With this "label bias," one should exclude a feature if its correlation with the proxy and its correlation with the true outcome have opposite signs, conditional on the other model features. This criterion is often satisfied when a feature is weakly correlated with the true outcome, and, additionally, that feature and the true outcome are both direct causes of the proxy outcome. For example, criminal behavior and geography may be weakly correlated and, due to patterns of police deployment, direct causes of one's arrest record—suggesting that excluding geography in criminal risk assessment will weaken an algorithm's performance in predicting arrest but will improve its capacity to predict actual crime.

INTRODUCTION

Risk assessments are central to the allocation of resources and the imposition of sanctions. In medicine, estimated health risks guide treatment decisions (*1*); in banking, default risk determines whether an applicant should be granted a loan (*2*); in education, the risk of noncompletion is an important factor for college admissions decisions (*3*); and in criminal justice, recidivism risk helps judges decide whether to detain or release a defendant while their cases proceed (*4*–*6*). Increasingly, the risk of these adverse events is estimated with the help of statistical algorithms. In training these algorithms, there is a widely shared view that the investigator should use as much data as is available to them (*7*–*9*). This view rests on the intuition that more information leads to predictions that are at least as good as those with less information: If the added data are informative in estimating risk, then they will improve the performance of the algorithm, and if the added data do not contain a helpful signal, then they will be discarded without hurting performance. Proponents of this view stress that feature importance in the predictive context neither requires nor implies a causal link between algorithmic inputs and predicted outcomes (*8*). Without the constraints of rigorous causal identification, it is argued that investigators can remain entirely atheoretical and simply hand all available data over to the predictive algorithm.

Here, we show how "label bias," present in virtually all real-world scenarios in which algorithms are deployed today, can invalidate this common rationale. Label bias occurs when the outcome of interest is not observed directly but is instead observed with systematic measurement error. For instance, although criminal risk assessment tools seek to estimate the risk of future criminal behavior, we typically only observe whether individuals are arrested or convicted of a crime. Similarly, tools used to estimate health risk often seek to divert resources to the patients with the most serious medical needs, but our observations are often limited to medical expenditures. The inclusion of additional features will in general improve an algorithm's prediction of the proxy label (e.g., arrest or medical expenditures), but in the presence of label bias, the additional information can decrease the quality of predictions for the true label (e.g, criminal behavior or medical need). Below, we formally demonstrate and empirically illustrate conditions under which the inclusion of additional features hurts the predictive performance on the true outcome of interest. Because researchers rarely have access to the true label, whether or not to include a particular feature often rests on unverifiable assumptions about the relationships that gave rise to the proxy label. The findings highlight that most predictive contexts require investigators to spend substantial time and care in developing a theoretical model of the underlying data generating process. The importance of creating such a model is often seen as something that is the exclusive domain of causal inference, but we highlight here that it is also important in predictive contexts.

Our study contributes to a burgeoning literature examining the use of algorithmic risk prediction in a variety of domains. These algorithms are frequently used to predict the risk of adverse events such as future criminal offending and failure to appear in court (*10*), the risk of child abuse (*11*–*13*), money laundering (*14*), students lagging behind in their learning (*15*), and the risk of nonpayment of loans (*2*). They are also used in situations where organizations or governments are deciding how to allocate scarce resources such as providing building permits (*16*), assigning students to schools (*17*), assigning high-risk patients to programs providing them more care (*18*), and determining who will receive kidney transplants (*19*). Further, corporations are currently using these tools to inform decisions about who receives information about housing advertisements (*20*) and employment opportunities (*21*). Algorithmic risk assessment tools can be better than humans at determining risk (*22*). However, scholars continue to critique these algorithms and study whether and under what conditions they can fairly and effectively be deployed in society (*23*–*26*).

In addition, our analysis builds on and contributes to a substantial body of literature examining the impact of label bias in statistical analyses. Prior work in the social sciences has long focused on the importance of measurement error for causal studies. Within this literature, a main focus has traditionally been on examining the importance of measurement error in the independent variable, which can, at best, attenuate the causal estimates [pp. 320–323 in (*27*)] and, at worst, bias the coefficients in ways that are difficult to predict (*28*). Less attention has been given to label bias (i.e., measurement error in the dependent variable), perhaps because it is often assumed that proxy labels differ from the true labels by random noise, in which case one can still

[1]Sociology and Social Policy, Harvard University, Cambridge, MA 02138, USA. [2]Stanford Law School, Stanford, CA 94305, USA. [3]Harvard Kennedy School, Cambridge, MA 02138, USA.
*Corresponding author. Email: michael_zangertishler@g.harvard.edu

obtain unbiased causal estimates [pp. 318–320 in (*27*)]. Existing research, however, suggests that there is a nonrandom relationship between the true and proxy labels across a variety of contexts, such as in the case of offending (i.e., actually committing a crime) and arrest (*29*). More recent contributions have considered the impact of such systematic errors in the labels. For example, Knox *et al.* (*30*) examine the potential for biases to arise in causal estimates when latent concepts that cannot be directly measured—such as political "ideology" and "democracy"—are approximated by proxy variables constructed from statistical models. Complementary work in computer science has examined the impact of label bias in a predictive setting. For instance, although predictive models may perform well on the proxy label, research has shown that they are not guaranteed to be accurate on the true label if the measurement error between the true and proxy label is non-random (*31*). Similarly, label bias can also reduce the fairness of these algorithms on the true label (*32*). When feasible, training predictions on the true label rather than a proxy has been shown to reduce racial inequities in algorithmic prediction and increase performance (*18*, *33*, *34*).

We build on these contributions by explicitly examining how the performance decrease from label bias interacts with the inclusion of additional input features into the model. To establish our results, we begin, in Methods, by deriving analytic conditions for when excluding features in a model trained to predict a proxy label is guaranteed to improve predictions of the true outcome of interest. We demonstrate and build intuition for these analytic results using a stylized example of estimating recidivism risk in the presence of label bias, where reoffense is the true label of interest and rearrest is the observed proxy. Then, in Results, we turn to two case studies. First, we consider partially synthetic recidivism data with real rearrest outcomes (the proxy label) and simulated reoffense outcomes (the true label). This setting resembles one that many researchers face in practice, where data on the true label are often prohibitively difficult or impossible to obtain. We show how different assumptions about how the true label relates to the observed proxy affect decisions about what features to include in the risk assessment model. Second, we consider a dataset from the health sciences. In targeting patients for high-risk care management programs, we rely on data by Obermeyer *et al.* (*18*), which contain, among other items, information on both the true label (health care need) and a proxy (health care spending). Using this dataset, we estimate the welfare costs of using a kitchen-sink predictive model instead of more judiciously selecting a model that accounts for label bias. We conclude in Discussion with a recap of our findings and suggest potential paths forward.

METHODS

A statistical condition for excluding features

To build intuition for how label bias impacts the choice of features in predictive models, we start with a simplified motivating example from the criminal justice context. In the United States, after an arrest, a judge will often decide whether or not to detain the arrested individual based on their estimated risk to public safety. In practice, this risk is commonly estimated using statistical risk assessments. The underlying risk models are trained using information about future arrests and convictions. However, arrests and convictions are not direct measures of public safety risks. Instead, they merely act as proxies, making these risk assessment tools susceptible to label bias.

In Fig. 1, we sketch the data-generating process for a stylized, linear structural equation model (SEM) (*35*) of arrests and behavior, where we treat arrests as the observed proxy for unobserved behavior, our true outcome of interest. The model produces synthetic data on individual-level behavior (B_0 and B_1) and arrest (A_0 and A_1) outcomes at two time periods ($t = 0$ and $t = 1$), as well as the neighborhood (Z) in which the individual resides. Arrests depend both on behavior and on neighborhood, reflecting the fact that people who engage in the same behavior may be arrested at different rates depending on where they live. For example, Beckett *et al.* (*36*) found that the geographic concentration of police resources in Seattle led to higher arrest rates for Black individuals delivering drugs compared to white individuals delivering drugs—where the true racial distribution of those delivering drugs was

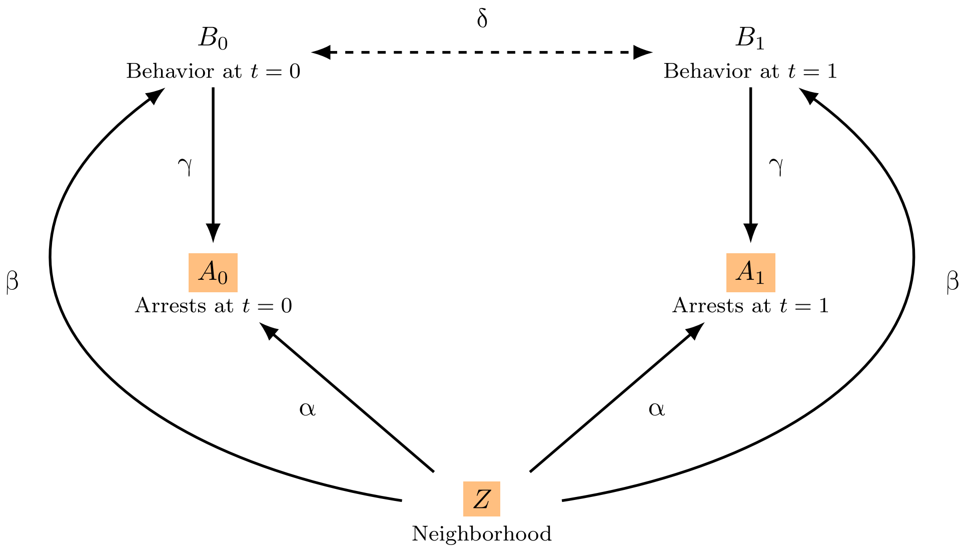

Fig. 1. The data-generating process for our stylized example of criminal behavior (true label) and arrest (proxy label). Observed variables are highlighted in orange.

estimated from survey data and ethnographic observations. Similarly, Cai *et al.* (*37*) found that the issuance of speeding tickets varied across neighborhoods even after adjusting for the true underlying incidence of speeding, as estimated by the movement of mobile phones.

In this SEM, all of the variables are normally distributed, with a mean of 0 and a variance of 1. We can thus interpret their values as representing the extent to which individuals differ from the population averages. In the case of neighborhood (Z), we can think of its value as denoting the level of police enforcement in that area. Further details about the model are provided in the Supplementary Materials.

Using synthetic data generated with this SEM, we train a "complex," kitchen-sink model to predict arrests at time $t = 1$ (A_1) based on arrests at time $t = 0$ (A_0) and neighborhood (Z). The more parsimonious, "simple" model bases its predictions only on arrests at time $t = 0$, omitting neighborhood. We now examine how the performance of the complex and simple models vary for different values of β, the parameter that describes the relationship between neighborhood and behavior, holding the other parameters fixed. For this simulation, we set $\alpha = \gamma = \delta = 0.4$, although the general pattern is largely invariant to this choice, as we describe in more detail below.

Across values of β, the left-hand panel of Fig. 2 shows that the complex model outperforms the simple model—in terms of root mean squared error (RMSE)—when evaluated on the proxy label. As expected, including more information reduces error when evaluated on the label used to train the models, a pattern that has traditionally motivated the inclusion of more features in predictive models. However, moving to the right-hand panel of Fig. 2, we see that the simple model outperforms the complex model on the true label for some values of β. In particular, the simple model outperforms the complex one for small values of β, corresponding to a weak relationship between neighborhood and behavior.

Our SEM illustrates a scenario in which simple models outperform more complex models due to the presence of label bias. To understand this result, imagine two individuals, both of whom have the same prior arrest record, but with only one of them living in a heavily policed neighborhood. Further assume that where one lives has little impact on criminal behavior (corresponding to small β), but that heavier policing increases the chance of being arrested for an offense. In this case, we can infer that the individual living in the heavily policed neighborhood engaged in past criminal activity less frequently than the individual living in the less heavily policed neighborhood. This is because fewer actual offenses are required to build a given arrest record in areas of high enforcement. Extrapolating from their past behavior, we would accordingly expect the individual in the heavily policed area to be less likely to engage in future criminal behavior. Thus, using information about one's neighborhood to predict future arrests (the proxy label) correctly tells us that the individual living in the heavily policed neighborhood is more likely to be rearrested, but it incorrectly suggests that individual is also more likely to engage in future criminal behavior (the true label). So, when predicting arrests as a proxy for behavior, it is better in this case to exclude information on one's neighborhood.

The SEM depicts a specific data-generating process, but the phenomenon we identify is generalizable. Theorem 1 and Corollary 1 below establish formal conditions under which this pattern is guaranteed to occur. Proofs for both results are straightforward, and are provided in the Supplementary Materials.

Theorem 1. Suppose Y and Y' are two arbitrary random variables with finite variance, where Y is the "true" outcome of interest and Y' is a proxy. For a random vector $X = (X_1, \ldots, X_k)$ and a random vector $Z = (Z_1, \ldots, Z_\ell)$, consider the estimators

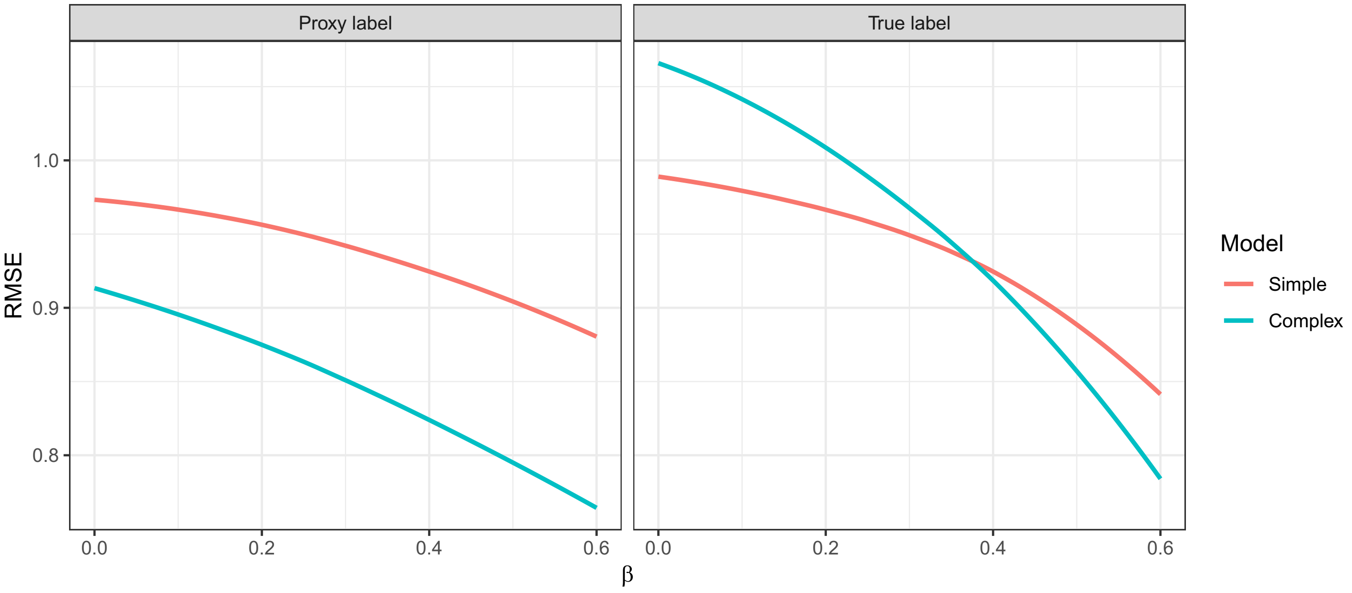

Fig. 2. Performance of simple and complex models trained to predict a proxy label, when evaluated on the proxy label (left) and the true label (right) for a range of β values. Whereas the complex model outperforms the simple model on the proxy label, the simple model outperforms the complex model on the true label for certain values of β. RMSE, root mean squared error.

$$\hat{Y}_{X,Z} = \mathbb{E}[Y'|X,Z] \text{ and}$$

$$\hat{Y}_X = \mathbb{E}[Y'|X]$$

where $\hat{Y}_{X,Z}$ is the "complex" estimator that uses all available features and $\hat{Y}_X$ is the simple estimator that omits Z. Then,

$$\mathbb{E}\left[\left(\hat{Y}_{X,Z} - Y\right)^2\right] - \mathbb{E}\left[\left(\hat{Y}_X - Y\right)^2\right] = \left(\mathbb{E}[\mathrm{Var}(Y'|X)] - \mathbb{E}[\mathrm{Var}(Y'|X,Z)]\right) - 2\mathbb{E}\left[\mathrm{Cov}\left(\hat{Y}_{X,Z}, Y|X\right)\right] \quad (1)$$

In particular,

$$\mathbb{E}\left[\left(\hat{Y}_{X,Z} - Y\right)^2\right] - \mathbb{E}\left[\left(\hat{Y}_X - Y\right)^2\right] \geq -2\mathbb{E}\left[\mathrm{Cov}\left(\hat{Y}_{X,Z}, Y|X\right)\right] \quad (2)$$

with strict inequality if $\hat{Y}_{X,Z} \neq \hat{Y}_X$.

In the setting of Theorem 1, one seeks to estimate a true outcome of interest Y and is choosing between two different estimators designed to predict the proxy label Y'. The first, complex estimator ($\hat{Y}_{X,Z}$) uses both X and Z to predict Y', whereas the second ($\hat{Y}_X$) uses only X. The theorem shows that if, conditional on X, the true label (Y) is negatively correlated with the complex estimator ($\hat{Y}_{X,Z}$), then the simple model generally outperforms the complex estimator—in terms of mean squared error—on the true outcome of interest. Intuitively, this result holds because the condition of the theorem means that the complex estimator goes in the "wrong" direction relative to the true outcome of interest.

If, alternatively, the true and proxy labels differ only by additive, independent noise, then Proposition 1 in the Supplementary Materials shows that including more information when predicting the proxy label will in general improve predictive performance on the true label. In the absence of systematic measurement error—including the case where there is no measurement error—the proposition confirms the conventional wisdom that more information is better.

To build further insight into this result, we consider the case where $\ell = 1$ (i.e., Z is a single random variable) and the complex estimator $\hat{Y}_{X,Z}$ is linear in Z. In this setting, Corollary 1 establishes a simpler condition under which performance increases by omitting information. Specifically, if, conditional on X, Z is positively correlated with true label Y but negatively correlated with the proxy label Y' (or vice versa), then omitting Z when predicting the proxy label will in general improve performance on the true outcome of interest.

Corollary 1. Consider the setting of Theorem 1 with $\ell = 1$. Suppose additionally that Z has finite variance and $\hat{Y}_{X,Z}$ is linear in Z, i.e., $\hat{Y}_{X,Z} = f(X) + cZ$ for some function f and a constant $c \in \mathbb{R}$. If $\hat{Y}_{X,Z} \neq \hat{Y}_X$ and either $\mathbb{E}[\mathrm{Cov}(Y,Z|X)] = 0$ or

$$\mathrm{sign}(\mathbb{E}[\mathrm{Cov}(Y,Z|X)]) = -\mathrm{sign}(\mathbb{E}[\mathrm{Cov}(Y',Z|X)])$$

then, $\mathbb{E}\left[\left(\hat{Y}_X - Y\right)^2\right] < \mathbb{E}\left[\left(\hat{Y}_{X,Z} - Y\right)^2\right]$.

The linearity assumption of Corollary 1 holds in a variety of settings. In particular, as described in the Supplementary Materials, it holds when Y', X, and Z are jointly multivariate normal, as is the case in our SEM above. To apply the corollary, one needs information on the correlations of Y and Z and of Y' and Z, conditional on X. The former involves directly observed quantities—the proxy label and the potential input features—and so, in practice, can be computed from the data. For our stylized SEM, we show in the Supplementary Materials that this correlation is positive for all (nondegenerate) parameter choices, meaning that neighborhood (Z) is positively correlated with future arrests (A_1), conditional on past arrests (A_0). The second conditional correlation we must consider when applying Corollary 1—the correlation between Y and Z, conditional on X—is not typically directly observed, as it depends on the true label Y. Understanding its sign thus involves assumptions about how the true label is related to the input features Z and X. For our SEM, we show in the Supplementary Materials that this correlation is negative for small values of β. That is, when β is small, neighborhood (Z) and future behavior (B_1) are negatively correlated conditional on past arrests (A_0). Intuitively, this is because A_0 is a collider—a variable caused by two other variables—and so when we fix its value, increasing Z requires decreasing B_0, which, in turn, decreases B_1. Thus, for small values of β, omitting neighborhood when predicting the proxy label improves performance on the true label, as shown in Fig. 3.

RESULTS

Case studies

To better understand the practical implications of our results, we now turn to two real-world datasets. The first allows us to further consider criminal risk assessments, adding additional realism to our stylized SEM above; the second dataset comes from the medical domain, where the goal of the risk assessment we consider is to identify patients with complex health care needs.

Criminal risk assessments

Continuing with our running example studying arrest and criminal behavior, we use data on individuals from a major U.S. county who were arrested for a felony offense between 2013 and 2019. For simplicity, we limit the sample to the 25,918 cases where the individuals' race was identified as either Black or non-Hispanic white. The dataset includes further details on each case, including information on the charges, the location, date and time of the incident, and the criminal history of the arrested individual. In addition, the dataset contains information on future rearrests, which we use as our proxy label for future offenses. Using these data, we fit simple and complex models trained on the proxy label (future arrests). We then examine model performance on the true label (future criminal offenses), which we simulate, as described below, because it is not directly observed. Our complex model includes three features: the age of the arrested individual, the number of times the individual was previously arrested, and whether or not the arrest occurred in a "high policing" area (i.e., a police district accounting for disproportionately high numbers of arrests). Our simple model includes age and number of past arrests, but not location information—similar to many commonly used criminal risk assessment tools.

This example mirrors many instances of label bias in the real world, as it is difficult—and perhaps impossible—to directly estimate the risk of true offending (*38*). This is partly because criminal

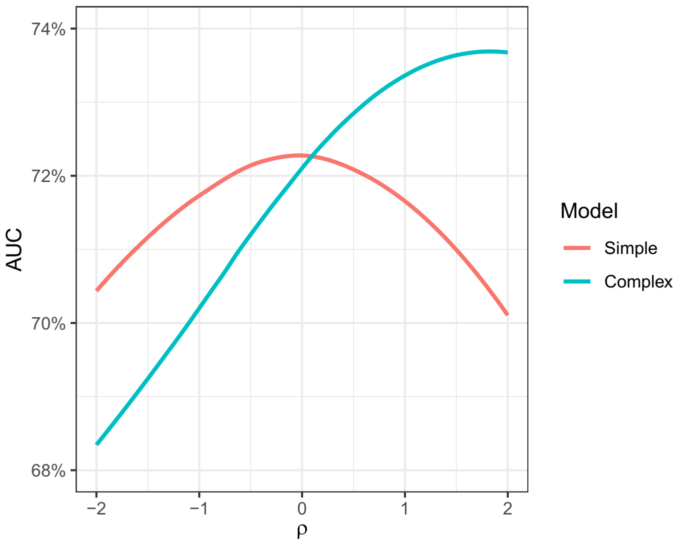

Fig. 3. Performance of simple (age and past arrests) and complex (age, past arrests, and neighborhood) models trained to predict future arrests (the proxy label), evaluated on future criminal behavior (the true label). Because the future criminal behavior is not directly observable, the plot shows results for synthetic outcomes generated under a range of data-generating processes parameterized by ρ, the hypothesized relationship between neighborhood and future criminal behavior, conditional on age, and past arrests.

behavior that is not reported to the police will not be included in administrative records. We thus simulate offending outcomes under a range of data-generating processes and then examine how assumptions about criminal behavior affect model performance after including or omitting location information. In particular, we parameterize these data-generating processes in terms of a fixed value $\rho \in \mathbb{R}$ describing the relationship between neighborhood and criminal behavior, conditional on age and past arrests. We then assume that each individual in our dataset commits a future offense with the following probability

$$\Pr(B_1 = 1) = \text{logit}^{-1}\left(-1 - \frac{1}{100} X_{\text{age}} + \frac{1}{2} A_0 + \rho Z \right)$$

where B_1 indicates future criminal behavior (our true label), X_{age} is the arrested individual's age, A_0 is the number of times they were previously arrested, and Z indicates whether the arrest took place in a high-policing area. The intercept and the coefficients for A_0 and X_{age} were selected to approximate the coefficients from a regression of future arrests on age and past arrests in our data.

On the basis of the data-generating process described above, we now evaluate the ability of our simple and complex risk assessment models to predict the synthetic true label, future criminal behavior. We evaluate model performance in terms of the area under the receiver operating characteristic curve (AUC), as the outcome is binary. AUC is a common measure of performance in the machine learning community when considering binary outcomes. Given a random individual who engaged in future criminal activity and a random individual who did not, the AUC of a risk assessment model is the probability that the model correctly identifies the individual in the pair who engaged in criminal activity. Our formal theoretical results are stated in terms of RMSE, but this example and our subsequent example show that the general pattern and intuition extend to other popular evaluation metrics.

Figure 3 shows that the simple model outperforms the complex model on the true label when ρ is negative, and the complex model outperforms the simple model when ρ is sufficiently positive. Given two arrested individuals who are the same age and have the same number of past arrests, negative values of ρ indicate that the individual who was arrested in the high-policing area is the less likely of the pair to engage in future criminal behavior. Accordingly, to the extent that one believes the hypothesized data-generating process with negative ρ is a sufficiently accurate description of criminal behavior, it is better to exclude neighborhood information when training risk assessment tools on the proxy label future arrests.

Identifying high-needs patients

We continue by applying our results to a well-known case of label bias in the literature, that of a commercial risk assessment tool relied on by health systems to target patients for "high-risk care management" programs (*18*). These programs seek to enroll patients with complex medical needs and subsequently provide them with a higher level of care. Because these programs are capacity constrained, the role of statistical risk assessments in this case is to accurately identify patients who would benefit the most from the additional care. In practice, the risk assessment algorithms are often designed to predict future medical expenditures, a proxy for medical need as the true outcome of interest. Analyzing these algorithms, Obermeyer *et al.* (*18*) conclude that, due to label bias, Black patients are less likely to be enrolled in the program than white patients with the same level of medical need. This is because unequal access to health care means that white individuals are more likely to seek medical treatment—and accordingly incur higher medical costs—than equally sick racial minorities.

Obermeyer *et al.* (*18*) highlight the importance of appropriately selecting the target of prediction and illustrate the accuracy and equity gains one can achieve by switching from predicting expenditures to a more direct measure of medical need. Here, we revisit the problem and investigate how the choice of risk factors used to identify patients affects enrollment decisions. To do so, we start with the data released by Obermeyer *et al.* (*18*), which include detailed information on patient demographics (sex, race, and age), current and future health, and past and future medical expenditures. To preserve patient confidentiality, variables in the released dataset were synthetically generated in a manner that ensures their conditional distributions approximate those in the original, unreleased dataset. We then train simple and complex models on the proxy label, future medical costs. Our complex model includes all information available at the time of the enrollment decision (i.e., patient demographics, current health, and past medical expenditures); our simple model includes only current health, excluding past medical costs and demographic variables. In this case, the equivalent of our parallel "neighborhood" variables are past expenditure and demographics variables. In the end, the complex model includes 150 predictors, and the simple model includes 128 predictors.

Next, we evaluate both models on their ability to predict whether a patient, in the subsequent year, is found to suffer from at least three chronic diseases—a measure of future health need identified by Obermeyer *et al.* (*18*). The left-hand panel of Fig. 4 shows the number of high-needs patients enrolled under the simple and complex models at different enrollment capacities, where the patients with

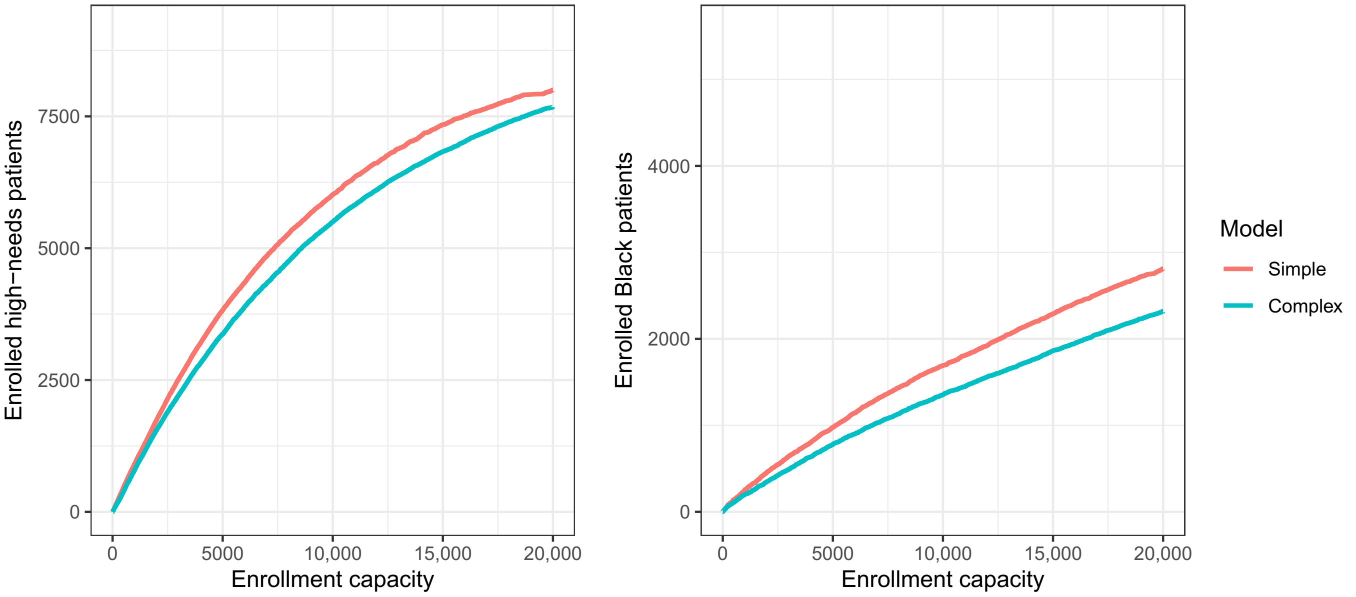

Fig. 4. Enrollment of high needs patients (left) and demographic composition of enrolled patients (right) under the simple and complex models for a range of program capacities.

highest estimated risk under the respective models are enrolled in the program. At each capacity level, the simple model outperforms the complex model in identifying more high-needs patients. In addition, as shown in the right-hand panel of Fig. 4, the simple model enrolls more Black patients than the complex model at every capacity level. This pattern stems from the simple model prioritizing patients with high expected medical needs over patients with high expected medical expenditures—the latter population being disproportionately white. Thus, if one only has access to a proxy label, then systematically excluding input features in a risk assessment tool can improve both the accuracy and equity of the instrument.

DISCUSSION

In building predictive models, the traditional guidance is to include all available information to maximize performance. However, as we have shown, a more judicious selection of features can lead to better model performance in the presence of label bias. Because the true label of interest is often not readily available, it raises the question of what examiners should and can do to mitigate the negative consequences from using a kitchen-sink model for prediction. The examples we have discussed highlight several approaches that vary in their appropriateness based on data availability and understanding of the underlying data-generating process.

Most directly, Obermeyer *et al.* (*18*) illustrate how some instances of label bias can be addressed simply by making a more concentrated effort to collect data on the true label of interest. If such an effort is generally possible but prohibitively costly, then investigators should consider whether the true label of interest can be obtained for a smaller subset of the population. This subset, even if it is not sufficiently large to train models predicting the true label, might still be used to explore how the selection of features affects model performance on the true label. If obtaining the true label is impossible, but investigators have access to a wealth of other features, one may simulate the true label of interest. In doing so, researchers should use their domain-specific knowledge to make reasonable assumptions about the relationship between the true label of interest and the features in question. We illustrated this process using felony offense data. Investigators need not constrain themselves to one particular relationship between the true label and the features but can instead assess the sensitivity of feature selection to label bias across a wide range of plausible assumptions. Last, investigators can make additional theoretical assumptions about the data-generating process to determine how label bias affects the choice of risk factors in a specific application—as we did in our health care example. As shown in that example, caution is particularly warranted for features that do not appear to be directly risk relevant. These features often yield little improvement on the true outcome of interest and raise the likelihood that performance may decrease or that their inclusion may exacerbate disparities.

More generally, our findings suggest—in contrast to conventional wisdom—that one cannot entirely divorce the predictive enterprise from theoretical considerations. Instead, a successful deployment of predictive tools often rests on the plausibility of the assumptions about the underlying processes that give rise to the observed data, highlighting the continued utility of domain-specific expertise in the predictive context.

Supplementary Materials

This PDF file includes:
Supplementary Text
References

REFERENCES AND NOTES

1. S. Mullainathan, Z. Obermeyer, Diagnosing physician error: A machine learning approach to low-value health care. *The Quarterly Journal of Economics* **137**, 679–727 (2022).
2. M. Leo, S. Sharma, K. Maddulety, Machine learning in banking risk management: A literature review. *Risks* **7**, 29 (2019).

3. L. Aulck, D. Nambi, N. Velagapudi, J. Blumenstock, J. West, Mining university registrar records to predict first-year undergraduate attrition. (International Educational Data Mining Society, 2019).
4. A. Chouldechova, Fair prediction with disparate impact: A study of bias in recidivism prediction instruments. *Big Data* **5**, 153–163 (2017).
5. J. Dressel, H. Farid, The accuracy, fairness, and limits of predicting recidivism. *Sci. Adv.* **4**, eaao5580 (2018).
6. Z. Lin, J. Jung, S. Goel, J. Skeem, The limits of human predictions of recidivism. *Sci. Adv.* **6**, eaaz0652 (2020).
7. R. Berk, *Machine Learning Risk Assessments in Criminal Justice Settings* (Springer, 2019).
8. C. F. Manski, Patient-centered appraisal of race-free clinical risk assessment. *Health Econ.* **31**, 2109–2114 (2022).
9. C. F. Manski, J. Mullahy, A. S. Venkataramani, Using measures of race to make clinical predictions: Decision making, patient health, and fairness. *Proc. Natl Acad. Sci. U.S.A.* **120**, e2303370120 (2023).
10. K. Imai, Z. Jiang, D. J. Greiner, R. Halen, S. Shin, Experimental evaluation of algorithm-assisted human decision-making: Application to pretrial public safety assessment. *J. R. Stat. Soc. Ser. A: Stat.Soc* **186**, 167–189 (2023).
11. A. Brown, A. Chouldechova, E. Putnam-Hornstein, A. Tobin, R. Vaithianathan, Toward algorithmic accountability in public services: A qualitative study of affected community perspectives on algorithmic decision-making in child welfare service, in *Proceedings of the 2019 CHI Conference on Human Factors in Computing Systems* (2019), pp. 1–12.
12. A. Chouldechova, D. Benavides-Prado, O. Fialko, R. Vaithianathan, A case study of algorithm-assisted decision making in child maltreatment hotline screening decisions, in *Conference on Fairness, Accountability and Transparency* (2018), pp. 134–148.
13. R. Shroff, Predictive analytics for city agencies: Lessons from children's services. *Big Data* **5**, 189–196 (2017).
14. Y. Zhang, P. Trubey, Machine learning and sampling scheme: An empirical study of money laundering detection. *Comput. Economics* **54**, 1043–1063 (2019).
15. L, Cattell, J. Bruch, Identifying students at risk using prior performance versus a machine learning algorithm (Technical report, U.S. Department of Education, Institute of Education Sciences, National Center for Education Evaluation and Regional Assistance, Regional Educational Laboratory Mid-Atlantic, 2021).
16. V. Mayer-Schönberger, K. Cukier, *Big Data: A Revolution That Will Transform How We Live, Work, and Think* (Houghton Mifflin Harcourt, 2013).
17. M. Allman, I. Ashlagi, I. Lo, J. Love, K. Mentzer, L. Ruiz-Setz, H. O'Connell, Designing school choice for diversity in the San Francisco Unified School District, in *Proceedings of the 23rd ACM Conference on Economics and Computation* (ACM, 2022), pp. 290–291.
18. Z. Obermeyer, B. Powers, C. Vogeli, S. Mullainathan, Dissecting racial bias in an algorithm used to manage the health of populations. *Science* **366**, 447–453 (2019).
19. J. J. Friedewald, C. J. Samana, B. L. Kasiske, A. K. Israni, D. Stewart, W. Cherikh, R. N. Formica, The kidney allocation system. *Surg. Clin.* **93**, 1395–1406 (2013).
20. T. Speicher, M. Ali, G. Venkatadri, F. N. Ribeiro, G. Arvanitakis, F. Benevenuto, K. P. Gummadi, P. Loiseau, A. Mislove, Potential for discrimination in online targeted advertising, in *Proceedings of the 1st Conference on Fairness, Accountability and Transparency* (ACM, 2018), pp 5–19.
21. A. Lambrecht, C. Tucker, Algorithmic bias? An empirical study of apparent gender-based discrimination in the display of stem career ads. *Manag. Sci.* **65**, 2966–2981 (2019).
22. S. Goel, R. Shroff, J. Skeem, C. Slobogin, *The Accuracy, Equity, and Jurisprudence of Criminal Risk Assessment*, in *Resarch Handbook on Big Data Law* (Edward Elgar Publishing, 2021), pp 9–28.
23. Angelina Wang, Sayash Kapoor, Solon Barocas, and Arvind Narayanan. Against predictive optimization: On the legitimacy of decision-making algorithms that optimize predictive accuracy, in *Proceedings of the 2023 ACM Conference on Fairness, Accountability, and Transparency* (ACM, 2023), pp. 626–626.
24. S. Corbett-Davies, J. Gaebler, H. Nilforoshan, R. Shroff, S. Goel, The measure and mismeasure of fairness. *J. Mach. Learn. Res*, (2023).
25. A. Chouldechova, A. Roth, A snapshot of the frontiers of fairness in machine learning. *Commun. ACM* **63**, 82–89 (2020).
26. A. Chohlas-Wood, M. Coots, S. Goel, J. Nyarko, Designing equitable algorithms. *Nat. Comput. Sci.* **3**, 601–610 (2023).
27. J. M. Wooldridge, *Introductory Econometrics: A Modern Approach* (Cengage Learning, 2015).
28. A. Chalfin, J. McCrary, Are U.S. Cities Underpoliced? Theory and evidence. *Rev. Econ. Stat.* **100**, 167–186 (2018).
29. R. Fogliato, A. Xiang, Z. Lipton, D. Nagin, A. Chouldechova, On the validity of arrest as a proxy for offense: Race and the likelihood of arrest for violent crimes, in *Proceedings of the 2021 AAAI/ACM Conference on AI, Ethics, and Society* (ACM, 2021), pp. 100–111.
30. D. Knox, C. Lucas, W. K. T. Cho, Testing causal theories with learned proxies. *Annu. Rev. Polit. Sci.* **25**, 419–441 (2022).
31. J. Wang, Y. Liu, C. Levy, Fair classification with group-dependent label noise, in *Proceedings of the 2021 ACM conference on Fairness, Accountability, and Transparency* (ACM, 2021), pp. 526–536.
32. R. Fogliato, A. Chouldechova, M. G'Sell, Fairness evaluation in presence of biased noisy labels, in *2020 International Conference on Artificial Intelligence and Statistics* (ACM, 2022), pp. 2325–2336.
33. E. Pierson, D. M. Cutler, J. Leskovec, S. Mullainathan, Z. Obermeyer, An algorithmic approach to reducing unexplained pain disparities in underserved populations. *Nat. Med.* **27**, 136–140 (2021).
34. S. Mullainathan, Z. Obermeyer, On the inequity of predicting A while hoping for B, in *AEA Papers and Proceedings* (AEA, 2021), vol. 111, pp. 37–42.
35. J. Pearl, Linear models: A useful "microscope" for causal analysis. *J. Causal Infer.* **1**, 155–170 (2013).
36. K. Beckett, K. Nyrop, L. Pfingst, Race, drugs, and policing: Understanding disparities in drug delivery arrests. *Crim.* **44**, 105–137 (2006).
37. W. Cai, J. Gaebler, J. Kaashoek, L. Pinals, S. Madden, S. Goel, Measuring racial and ethnic disparities in traffic enforcement with large-scale telematics data. *PNAS Nexus* **1**, pgac144 (2022).
38. A. D. Biderman, A. J. Reiss Jr., On exploring the "dark figure" of crime. *Ann. Am. Acad. Pol. Soc. Sci.* **374**, 1–15 (1967).
39. S. Wright, Systems of mating. I. The biometric relations between parent and offspring. *Genetics* **6**, 111–123 (1921).
40. M. L. Eaton, *Multivariate Statistics: A Vector Space Approach* (John Wiley & Sons Inc., 1983).

Acknowledgments: We thank A. Feller, J. Gaebler, T. Gillis, J. Grossman, J. Roth, and C. Winship for helpful feedback and conversations. **Funding:** The authors acknowledge that they received no funding in support of this research. **Author contributions:** All authors contributed to all aspects of the paper, including conceptualization, analysis, and writing. **Competing interests:** The authors declare that they have no competing interests. **Data and materials availability:** All data needed to evaluate the conclusions in the paper are present in the paper and/or the Supplementary Materials. Data and code are available at the Harvard Dataverse (https://doi.org/10.7910/DVN/MYSQ2Q).

Submitted 21 May 2023
Accepted 21 February 2024
Published 29 March 2024
10.1126/sciadv.adi8411
