# Supplementary material for: Risk scores, label bias, and everything but the kitchen sink
Source: Sci Adv. 2024 Mar 29;10(13):eadi8411. doi: 10.1126/sciadv.adi8411 (PMC10980258; doi:10.1126/sciadv.adi8411)
Supplement: Supplementary file 2 — Supplementary Text References [file sciadv.adi8411_sm.pdf]

Science Advances
AAAS

Supplementary Materials for

Risk scores, label bias, everything but the kitchen sink

Michael Zanger-Tishler *et al.*

Corresponding author: Michael Zanger-Tishler, michael_zangertishler@g.harvard.edu

Sci. Adv. **10**, eadi8411 (2024)
DOI: 10.1126/sciadv.adi8411

This PDF file includes:

Supplementary Text
References

A Proof of Theorem 1

To start, note that for any square-integrable random variable $\hat{Y}$,

$$\mathbb{E}\left[\left(\hat{Y}-Y\right)^2\right]=\mathbb{E}\left[Y^2\right]+\mathbb{E}\left[\hat{Y}^2\right]-2\mathbb{E}\left[Y\cdot\hat{Y}\right].$$

Since Y' is square-integrable by assumption, so are $\hat{Y}_{X,Z}$ and $\hat{Y}_X$ (by the law of total variance), and so,

$$\begin{aligned}
\mathbb{E}&\left[\left(\hat{Y}_{X,Z}-Y\right)^2\right]-\mathbb{E}\left[\left(\hat{Y}_{X}-Y\right)^2\right]\\
&=\mathbb{E}\left[\hat{Y}_{X,Z}^2\right]-\mathbb{E}\left[\hat{Y}_{X}^2\right]+2\Big(\mathbb{E}\left[Y\cdot\hat{Y}_X\right]-\mathbb{E}\left[Y\cdot\hat{Y}_{X,Z}\right]\Big)\\
&=\mathrm{Var}\left(\hat{Y}_{X,Z}\right)-\mathrm{Var}\left(\hat{Y}_{X}\right)+2\Big(\mathbb{E}\left[Y\cdot\hat{Y}_X\right]-\mathbb{E}\left[Y\cdot\hat{Y}_{X,Z}\right]\Big)\\
&=\mathbb{E}\left[\mathrm{Var}(Y'\mid X)\right]-\mathbb{E}\left[\mathrm{Var}(Y'\mid X,Z)\right]+2\Big(\mathbb{E}\left[Y\cdot\hat{Y}_X\right]-\mathbb{E}\left[Y\cdot\hat{Y}_{X,Z}\right]\Big),
\end{aligned}\tag{1}$$

where the penultimate line follows from the fact that $\mathbb{E}\left[\hat{Y}_{X,Z}\right]=\mathbb{E}\left[\hat{Y}_X\right]=\mathbb{E}[Y']$, and the last line follows from the law of total variance. Now,

$$\begin{aligned}
\mathbb{E}\left[Y\cdot\hat{Y}_X\right]-\mathbb{E}\left[Y\cdot\hat{Y}_{X,Z}\right]&=\mathbb{E}\left[\mathbb{E}\left[Y\cdot\hat{Y}_X\mid X\right]-\mathbb{E}\left[Y\cdot\hat{Y}_{X,Z}\mid X\right]\right]\\
&=\mathbb{E}\left[\hat{Y}_X\cdot\mathbb{E}\left[Y\mid X\right]-\mathbb{E}\left[Y\cdot\hat{Y}_{X,Z}\mid X\right]\right]\\
&=\mathbb{E}\left[\mathbb{E}\left[\hat{Y}_{X,Z}\mid X\right]\cdot\mathbb{E}\left[Y\mid X\right]-\mathbb{E}\left[Y\cdot\hat{Y}_{X,Z}\mid X\right]\right]\\
&=-\mathbb{E}\left[\mathrm{Cov}\left(\hat{Y}_{X,Z},Y\mid X\right)\right],
\end{aligned}\tag{2}$$

where we repeatedly applied the law of iterated expectations, and used the fact that $\hat{Y}_X$ is measurable with respect to X in the second equality. Eqs. (1) and (2) together establish Eq. (1) in the theorem statement.

Eq. (2) in the theorem statement now follows immediately, since

$$\begin{aligned}
\mathbb{E}\left[\mathrm{Var}(Y'\mid X,Z)\right]&=\mathbb{E}\left[\mathbb{E}\left[\left(Y'-\hat{Y}_{X,Z}\right)^2\mid X,Z\right]\right]\\
&\leq\mathbb{E}\left[\mathbb{E}\left[\left(Y'-\hat{Y}_{X}\right)^2\mid X,Z\right]\right]\\
&=\mathbb{E}\left[\mathrm{Var}(Y'\mid X)\right],
\end{aligned}$$

where the inequality is strict if $\hat{Y}_{X,Z}\neq\hat{Y}_X$, establishing the result. □

B Proof of Corollary 1

By Theorem 1, it is sufficient to show that $\mathbb{E}\left[\mathrm{Cov}\left(\hat{Y}_{X,Z},Y\mid X\right)\right]\leq 0$. We start by noting that

$$\begin{aligned}
\mathbb{E}\left[\mathrm{Cov}\left(\hat{Y}_{X,Z},Y\mid X\right)\right]&=\mathbb{E}\left[\mathrm{Cov}\left(f(X)+cZ,Y\mid X\right)\right]\\
&=c\cdot\mathbb{E}\left[\mathrm{Cov}(Y,Z\mid X)\right].
\end{aligned}$$

Now, if $\mathbb{E}[\mathrm{Cov}(Y, Z \mid X)] = 0$, then the result follows immediately. If $\mathbb{E}[\mathrm{Cov}(Y, Z \mid X)] \neq 0$, then by the assumption of the theorem,

$$\mathrm{sign}\left(\mathbb{E}\left[\mathrm{Cov}\left(\hat{Y}_{X,Z}, Y \mid X\right)\right]\right) = -\mathrm{sign}\left(c \cdot \mathbb{E}\left[\mathrm{Cov}\left(Y', Z \mid X\right)\right]\right). \tag{3}$$

Now, by repeatedly applying the law of iterated expectations, we have

$$\begin{aligned}
\mathbb{E}\left[Z \cdot Y' \mid X\right] &= \mathbb{E}\left[\mathbb{E}\left[Z \cdot Y' \mid X, Z\right] \mid X\right] \\
&= \mathbb{E}\left[Z \cdot \mathbb{E}\left[Y' \mid X, Z\right] \mid X\right] \\
&= \mathbb{E}\left[Z \cdot \hat{Y}_{X,Z} \mid X\right] \\
&= f(X) \cdot \mathbb{E}[Z \mid X] + c \cdot \mathbb{E}\left[Z^2 \mid X\right].
\end{aligned}$$

Similarly, we have

$$\begin{aligned}
\mathbb{E}[Y' \mid X] &= \mathbb{E}[\mathbb{E}[Y' \mid X, Z] \mid X] \\
&= \mathbb{E}[\hat{Y}_{X,Z} \mid X] \\
&= f(X) + c \cdot \mathbb{E}[Z \mid X].
\end{aligned}$$

Putting the above together, we get

$$\begin{aligned}
\mathrm{Cov}\left(Y', Z \mid X\right) &= \mathbb{E}\left[Z \cdot Y' \mid X\right] - \mathbb{E}[Y' \mid X] \cdot \mathbb{E}[Z \mid X] \\
&= c \cdot \left(\mathbb{E}\left[Z^2 \mid X\right] - \mathbb{E}\left[Z \mid X\right]^2\right) \\
&= c \cdot \mathrm{Var}(Z \mid X).
\end{aligned}$$

Finally, by Eq. (3),

$$\begin{aligned}
\mathrm{sign}\left(\mathrm{Cov}\left(\hat{Y}_{X,Z}, Y \mid X\right)\right) &= -\mathrm{sign}\left(c^2 \cdot \mathrm{Var}(Z \mid X)\right) \\
&\leq 0,
\end{aligned}$$

establishing the result.

□

C Kitchen-Sink Models and Independent Noise

When the proxy label Y' and the true label Y simply differ by additive, independent noise, then it is advantageous to use all available information when constructing risk scores. The following proposition formalizes this statement.

Proposition 1 *In the setting of Theorem 1, suppose $Y' = Y + S$ where $S \perp\!\!\!\perp X, Z$. Then*

$$\mathbb{E}\left[\left(\hat{Y}_{X,Z} - Y\right)^2\right] \leq \mathbb{E}\left[\left(\hat{Y}_X - Y\right)^2\right].$$

Proof. First note that

$$\begin{aligned}\hat{Y}_{X,Z} &= \mathbb{E}[Y \mid X, Z] + \mathbb{E}[S \mid X, Z] \\ &= \mathbb{E}[Y \mid X, Z] + \mathbb{E}[S],\end{aligned}$$

where the second equality uses the independence assumption. Similarly,

$$\begin{aligned}\hat{Y}_{X} &= \mathbb{E}[Y \mid X] + \mathbb{E}[S \mid X] \\ &= \mathbb{E}[Y \mid X] + \mathbb{E}[S].\end{aligned}$$

Now, using the notation $Y_{X,Z} = \mathbb{E}[Y \mid X, Z]$ and $Y_X = \mathbb{E}[Y \mid X]$, we have

$$\begin{aligned}&\mathbb{E}\left[\left(\hat{Y}_{X,Z} - Y\right)^2\right] - \mathbb{E}\left[\left(\hat{Y}_{X} - Y\right)^2\right] \\ &\quad= \mathbb{E}\left[\left(Y_{X,Z} - Y + \mathbb{E}[S]\right)^2\right] - \mathbb{E}\left[\left(Y_{X} - Y + \mathbb{E}[S]\right)^2\right] \\ &\quad= \mathbb{E}\left[\left(Y_{X,Z} - Y\right)^2\right] - \mathbb{E}\left[\left(Y_{X} - Y\right)^2\right] + 2\mathbb{E}[S]\big(\mathbb{E}[Y_{X,Z} - Y] - \mathbb{E}[Y_X - Y]\big) \\ &\quad= \mathbb{E}\left[\left(Y_{X,Z} - Y\right)^2\right] - \mathbb{E}\left[\left(Y_{X} - Y\right)^2\right] \\ &\quad= \mathbb{E}\left[\mathbb{E}\left[\left(Y_{X,Z} - Y\right)^2 \mid X, Z\right]\right] - \mathbb{E}\left[\mathbb{E}\left[\left(Y_{X} - Y\right)^2 \mid X, Z\right]\right],\end{aligned}$$

where the third equality follows from the fact that $\mathbb{E}[Y_{X,Z}] = \mathbb{E}[Y_X] = \mathbb{E}[Y]$, and the last equality follows from the law of iterated expectations. Finally, since

$$\arg\min_c \mathbb{E}\left[\left(c - Y\right)^2 \mid X, Z\right] = Y_{X,Z},$$

we have that

$$\mathbb{E}\left[\left(Y_{X,Z} - Y\right)^2 \mid X, Z\right] - \mathbb{E}\left[\left(Y_{X} - Y\right)^2 \mid X, Z\right] \leq 0,$$

establishing the result.

□

D A Stylized Model of Arrest and Behavior

We formally describe and analyze the SEM depicted in Figure 1. Our model has three independent exogenous variables $U_Z = \mathrm{N}(0, \sigma_Z^2)$, $U_{A_0} = \mathrm{N}(0, \sigma_A^2)$, and $U_{A_1} = \mathrm{N}(0, \sigma_A^2)$. We additionally have two correlated exogenous variables $U_{B_0} = \mathrm{N}(0, \sigma_B^2)$ and $U_{B_1} = \mathrm{N}(0, \sigma_B^2)$ that are independent of the first three, with $\mathrm{Cov}(U_{B_0}, U_{B_1}) = \delta \geq 0$. Now, for non-negative constants α, β, and γ, the key variables in the model are generated by the following linear structural equations:

$$\begin{aligned}Z &= U_Z, \\ B_0 &= \beta Z + U_{B_0}, \\ B_1 &= \beta Z + U_{B_1}, \\ A_0 &= \alpha Z + \gamma B_0 + U_{A_0}, \\ A_1 &= \alpha Z + \gamma B_1 + U_{A_1}.\end{aligned} \tag{4}$$

We set the variances of the exogenous variables (σ_Z^2, σ_A^2, and σ_B^2) in a manner that ensures that the remaining variables (Z, B_0, B_1, A_0, and A_1) are standardized, meaning they have mean 0 and variance 1—we show how to do this below. We can thus interpret their values as representing the extent to which individuals differ from the population averages. In the case of neighborhood (Z), we can think of its value as denoting the level of police enforcement in an area.

To start, we set $\sigma_Z^2 = 1$, which ensures $\mathrm{Var}(Z) = 1$. Now, since $Z \perp\!\!\!\perp U_{B_0}$, we have that $\mathrm{Var}(B_0) = \beta^2 + \sigma_B^2$. Consequently, setting $\sigma_B^2 = 1 - \beta^2$ ensures that $\mathrm{Var}(B_0) = 1$ (and, similarly, that $\mathrm{Var}(B_1) = 1$). Finally, as above, $\mathrm{Var}(A_0) = \alpha^2 + \gamma^2 + \sigma_A^2 + 2\alpha\gamma\mathrm{Cov}(Z, B_0)$. One especially nice aspect of linear graphical models is that the covariance between any two variables can be immediately computed from the edge weights via the the Wright rules (*35, 39*). Specifically, when the nodes are standardized to have variance 1, then the covariance between any two variables in the graph is the sum, over all d-connected paths between the variables, of the product of the edge weights along the path. A path is d-connected if it does not pass through any colliders (i.e., nodes with head-to-head arrows along the path). To compute $\mathrm{Cov}(Z, B_0)$, observe that the only d-connected path between Z and B_0 is the direct path from Z to B_0, having edge weight β. As a result, $\mathrm{Cov}(Z, B_0) = \beta$, meaning that setting $\sigma_A^2 = 1 - \alpha^2 - \gamma^2 - 2\alpha\beta\gamma$ ensures that A_0 (and, analogously, A_1) have unit variance. Recapping, we have

$$\begin{aligned} \sigma_Z^2 &= 1, \\ \sigma_B^2 &= 1 - \beta^2, \\ \sigma_A^2 &= 1 - \alpha^2 - \gamma^2 - 2\alpha\beta\gamma. \end{aligned} \tag{5}$$

Our model is thus described by the four non-negative parameters α, β, γ, and δ, depicted as edge weights in Figure 1, with the constraint that the quantities in Eq. (5) are non-negative. Those constraints in turn imply that the parameters are each less than or equal to 1.

Our theoretical results in Theorem 1 and Corollary 1 require understanding the conditional distributions of model features. For multivariate normal random variables, these conditional distributions can be computed analytically (*40*), allowing us to examine properties of our motivating SEM in more depth. Specifically, suppose $\mathbf{W}$ is a k-dimensional multivariate normal random variable with mean $\boldsymbol{\mu}$ and covariance $\boldsymbol{\Sigma}$, which we partition into into its first q components and its remaining $k - q$ components: $\mathbf{W} = [\mathbf{W_1}, \mathbf{W_2}]$. Further suppose we accordingly partition $\boldsymbol{\mu}$ and $\boldsymbol{\Sigma}$ into its components:

$$\begin{aligned} \boldsymbol{\mu} &= \begin{bmatrix} \boldsymbol{\mu_1} \\ \boldsymbol{\mu_2} \end{bmatrix} \text{ with sizes } \begin{bmatrix} q \times 1 \\ (k-q) \times 1 \end{bmatrix}, \\ \boldsymbol{\Sigma} &= \begin{bmatrix} \boldsymbol{\Sigma_{11}} & \boldsymbol{\Sigma_{12}} \\ \boldsymbol{\Sigma_{21}} & \boldsymbol{\Sigma_{22}} \end{bmatrix} \text{ with sizes } \begin{bmatrix} q \times q & q \times (k-q) \\ (k-q) \times q & (k-q) \times (k-q) \end{bmatrix}. \end{aligned}$$

Then the distribution of $\mathbf{W_1}$ conditional on $\mathbf{W_2}$ is multivariate normal with mean

$$\boldsymbol{\mu_1} + \boldsymbol{\Sigma_{12}}\boldsymbol{\Sigma_{22}}^{-1}(\mathbf{W_2} - \boldsymbol{\mu_2})$$

and covariance

$$\boldsymbol{\Sigma_{11}} - \boldsymbol{\Sigma_{12}}\boldsymbol{\Sigma_{22}}^{-1}\boldsymbol{\Sigma_{21}}.$$

As a result, the linearity assumption of Corollary 1 is satisfied for multivariate normal random variables. In particular, in our motivating example, the conditional distribution of A_1 given A_0 and Z is

normal, with

$$\begin{aligned}\mathbb{E}[A_1 \mid A_0, Z] &= \begin{bmatrix}\sigma_{A_1A_0} & \sigma_{A_1Z}\end{bmatrix} \begin{bmatrix}1 & \sigma_{A_0Z} \\ \sigma_{A_0Z} & 1\end{bmatrix}^{-1} \begin{bmatrix}A_0 \\ Z\end{bmatrix} \\ &= \frac{1}{1-\sigma^2_{A_0Z}} \begin{bmatrix}\sigma_{A_1A_0} & \sigma_{A_1Z}\end{bmatrix} \begin{bmatrix}1 & -\sigma_{A_0Z} \\ -\sigma_{A_0Z} & 1\end{bmatrix} \begin{bmatrix}A_0 \\ Z\end{bmatrix} \\ &= \frac{\sigma_{A_1A_0} - \sigma_{A_1Z} \cdot \sigma_{A_0Z}}{1-\sigma^2_{A_0Z}} A_0 + \frac{\sigma_{A_1Z} - \sigma_{A_1A_0} \cdot \sigma_{A_0Z}}{1-\sigma^2_{A_0Z}} Z,\end{aligned}$$

where the σ notation denotes the covariance of the indexed random variables.

Further, the conditional distribution of (A_1, Z) given A_0 is likewise multivariate normal, with covariance matrix

$$\begin{aligned}\begin{bmatrix}1 & \sigma_{A_1Z} \\ \sigma_{A_1Z} & 1\end{bmatrix} - \begin{bmatrix}\sigma_{A_1A_0} \\ \sigma_{A_0Z}\end{bmatrix} \begin{bmatrix}\sigma_{A_1A_0} & \sigma_{A_0Z}\end{bmatrix} &= \begin{bmatrix}1 & \sigma_{A_1Z} \\ \sigma_{A_1Z} & 1\end{bmatrix} - \begin{bmatrix}\sigma^2_{A_1A_0} & \sigma_{A_1A_0} \cdot \sigma_{A_0Z} \\ \sigma_{A_1A_0} \cdot \sigma_{A_0Z} & \sigma^2_{A_0Z}\end{bmatrix} \\ &= \begin{bmatrix}1 - \sigma^2_{A_1A_0} & \sigma_{A_1Z} - \sigma_{A_1A_0} \cdot \sigma_{A_0Z} \\ \sigma_{A_1Z} - \sigma_{A_1A_0} \cdot \sigma_{A_0Z} & 1 - \sigma^2_{A_0Z}\end{bmatrix}.\end{aligned}$$

Consequently,

$$\mathrm{Cov}(A_1, Z \mid A_0) = \sigma_{A_1Z} - \sigma_{A_1A_0} \cdot \sigma_{A_0Z}, \tag{6}$$

and, analogously, we have that

$$\mathrm{Cov}(B_1, Z \mid A_0) = \sigma_{B_1Z} - \sigma_{B_1A_0} \cdot \sigma_{A_0Z}. \tag{7}$$

As above, we can compute the covariances in Eqs. (6) and (7) via the Wright rules. For example, as seen in Figure 1, there are two d-connected paths between Z and A_0: the direct connection with edge weight α; and the path through B_0, with product of edge weights $\beta\gamma$. Consequently, $\mathrm{Cov}(Z, A_0) = \alpha + \beta\gamma$. This procedure allows us to compute all of the terms appearing on the right-hand side of Eqs. (6) and (7), yielding:

$$\begin{aligned}\sigma_{A_0Z} &= \alpha + \beta\gamma \\ \sigma_{A_1Z} &= \alpha + \beta\gamma \\ \sigma_{B_1Z} &= \beta \\ \sigma_{A_1A_0} &= \alpha^2 + 2\alpha\beta\gamma + \beta^2\gamma^2 + \gamma^2\delta \\ \sigma_{B_1A_0} &= \alpha\beta + \beta^2\gamma + \gamma\delta.\end{aligned} \tag{8}$$

Leveraging the above, we now show that $\mathrm{Cov}(A_1, Z \mid A_0) \geq 0$, meaning that neighborhood is positively correlated with future arrests, conditional on past arrests. To see this, first note that

$$\begin{aligned}\delta &= \mathrm{Cov}(U_{B_0}, U_{B_1}) \\ &\leq \sigma^2_B \\ &= 1 - \beta^2,\end{aligned}$$

and so $\beta^2 + \delta \leq 1$. Now,

$$\begin{aligned}
\mathrm{Cov}(A_1, Z \mid A_0) &= \sigma_{A_1 Z} - \sigma_{A_1 A_0} \cdot \sigma_{A_0 Z} \\
&= \alpha + \beta\gamma - (\alpha + \beta\gamma) \cdot (\alpha^2 + 2\alpha\beta\gamma + \beta^2\gamma^2 + \gamma^2\delta) \\
&= (\alpha + \beta\gamma) \cdot (1 - \alpha^2 - 2\alpha\beta\gamma - \beta^2\gamma^2 - \gamma^2\delta) \\
&= (\alpha + \beta\gamma) \cdot (1 - \alpha^2 - 2\alpha\beta\gamma - \gamma^2(\beta^2 + \delta)) \\
&\geq (\alpha + \beta\gamma) \cdot (1 - \alpha^2 - 2\alpha\beta\gamma - \gamma^2) \\
&= (\alpha + \beta\gamma) \cdot \sigma_A^2 \\
&\geq 0,
\end{aligned}$$

where the first inequality follows from the fact that $\beta^2 + \delta \leq 1$.

Next we consider $\mathrm{Cov}(B_1, Z \mid A_0)$, and note that

$$\begin{aligned}
\mathrm{Cov}(B_1, Z \mid A_0) &= \sigma_{B_1 Z} - \sigma_{B_1 A_0} \cdot \sigma_{A_0 Z} \\
&= \beta - (\alpha\beta + \beta^2\gamma + \gamma\delta) \cdot (\alpha + \beta\gamma).
\end{aligned}$$

In particular, when $\beta = 0$, meaning that neighborhood does not impact behavior, then

$$\mathrm{Cov}(B_1, Z \mid A_0) = -\alpha\gamma\delta.$$

In other words, when neighborhood does not impact behavior (i.e., when $\beta = 0$), neighborhood is negatively correlated with future behavior conditional on past arrests. (And, by the above, neighborhood is always positively correlated with future arrests conditional on past arrests.) By Corollary 1, it is thus better in this case to base predictions of future behavior solely on past arrests, excluding neighborhood, as we see in Figure 2.

REFERENCES AND NOTES

1. S. Mullainathan, Z. Obermeyer, Diagnosing physician error: A machine learning approach to low-value health care. *The Quarterly Journal of Economics* **137**, 679–727 (2022).

2. M. Leo, S. Sharma, K. Maddulety. Machine learning in banking risk management: A literature review. *Risks* **7**, 29 (2019).

3. L. Aulck, D. Nambi, N. Velagapudi, J. Blumenstock, J. West, Mining university registrar records to predict first-year undergraduate attrition. (International Educational Data Mining Society, 2019).

4. A. Chouldechova, Fair prediction with disparate impact: A study of bias in recidivism prediction instruments. *Big Data* **5**, 153–163 (2017).

5. J. Dressel, H. Farid, The accuracy, fairness, and limits of predicting recidivism *Sci. Adv.* **4** eaao5580 (2018).

6. Z. Lin, J. Jung, S. Goel, J. Skeem, The limits of human predictions of recidivism *Sci. Adv.* **6**, eaaz0652 (2020).

7. R. Berk, *Machine Learning Risk Assessments in Criminal Justice Settings* (Springer, 2019).

8. C. F. Manski, Patient-centered appraisal of race-free clinical risk assessment. *Health Econ.* **31**, 2109–2114 (2022).

9. C. F. Manski, J. Mullahy, A. S. Venkataramani, Using measures of race to make clinical predictions: Decision making, patient health, and fairness. *Proc. Natl Acad. Sci.* **120**, e2303370120 (2023).

10. K. Imai, Z. Jiang, D. J. Greiner, R. Halen, S. Shin, Experimental evaluation of algorithm-assisted human decision-making: Application to pretrial public safety assessment. *J. R. Stat. Soc. Ser. A: Stat.Soc* **186**, 167–189 (2023).

11. A. Brown, A. Chouldechova, E. Putnam-Hornstein, A. Tobin, R. Vaithianathan, Toward algorithmic accountability in public services: A qualitative study of affected community perspectives on algorithmic decision-making in child welfare service, in *Proceedings of the 2019 CHI Conference on Human Factors in Computing Systems* (2019), pp. 1–12.

12. A. Chouldechova, D. Benavides-Prado, O. Fialko, R. Vaithianathan, A case study of algorithm-assisted decision making in child maltreatment hotline screening decisions, in *Conference on Fairness, Accountability and Transparency* (2018), pp. 134–148.

13. R. Shroff, Predictive analytics for city agencies: Lessons from children's services. *Big Data* **5**, 189–196 (2017).

14. Y. Zhang, P. Trubey, Machine learning and sampling scheme: An empirical study of money laundering detection. *Comput. Economics* **54**, 1043–1063 (2019).

15. L, Cattell, J. Bruch, Identifying students at risk using prior performance versus a machine learning algorithm (Technical report, U.S. Department of Education, Institute of Education Sciences, National Center for Education Evaluation and Regional Assistance, Regional Educational Laboratory Mid-Atlantic, 2021).

16. V. Mayer-Schönberger, K. Cukier, *Big Data: A Revolution That Will Transform How We Live, Work, and Think* (Houghton Mifflin Harcourt, 2013).

17. M. Allman, I. Ashlagi, I. Lo, J. Love, K. Mentzer, L. Ruiz-Setz, H. O'Connell, Designing school choice for diversity in the San Francisco Unified School District, in *Proceedings of the 23rd ACM Conference on Economics and Computation* (ACM, 2022), pp. 290–291.

18. Ziad Obermeyer, Brian Powers, Christine Vogeli, and Sendhil Mullainathan. Dissecting racial bias in an algorithm used to manage the health of populations. *Science* **366**, 447–453 (2019).

19. John J Friedewald, Ciara J Samana, Bertram L Kasiske, Ajay K Israni, Darren Stewart, Wida Cherikh, and Richard N Formica. The kidney allocation system. *Surg. Clin.* **93**, 1395–1406 (2013).

20. T. Speicher, M. Ali, G. Venkatadri, F. N. Ribeiro, G. Arvanitakis, F. Benevenuto, K. P. Gummadi, P. Loiseau, A. Mislove, Potential for discrimination in online targeted advertising, in *Proceedings of the 1st Conference on Fairness, Accountability and Transparency* (ACM, 2018), pp 5–19.

21. A. Lambrecht, C. Tucker, Algorithmic bias? An empirical study of apparent gender-based discrimination in the display of stem career ads. *Manag. Sci.* **65**, 2966–2981 (2019).

22. S. Goel, R. Shroff, J. Skeem, C. Slobogin, *The Accuracy, Equity, and Jurisprudence of Criminal Risk Assessment*, in *Research Handbook on Big Data Law* (Edward Elgar Publishing, 2021), pp 9–28.

23. Angelina Wang, Sayash Kapoor, Solon Barocas, and Arvind Narayanan. Against predictive optimization: On the legitimacy of decision-making algorithms that optimize predictive accuracy, in *Proceedings of the 2023 ACM Conference on Fairness, Accountability, and Transparency* (ACM, 2023), pp. 626–626.

24. S. Corbett-Davies, J. Gaebler, H. Nilforoshan, R. Shroff, S. Goel, The measure and mismeasure of fairness. *J. Mach. Learn. Res*, 2023.

25. A. Chouldechova, A. Roth, A snapshot of the frontiers of fairness in machine learning. *Commun. ACM* **63**, 82–89 (2020).

26. A. Chohlas-Wood, M. Coots, S. Goel, J. Nyarko, Designing equitable *algorithms. Nat. Comput. Sci.* **3**, 601–610 (2023).

27. J. M. Wooldridge, *Introductory Econometrics: A Modern Approach* (Cengage Learning, 2015).

28. A. Chalfin, J. McCrary, Are U.S. Cities Underpoliced? Theory and evidence. *Rev. Econ. Stat.* **100**, 167–186 (2018).

29. R. Fogliato, A. Xiang, Z. Lipton, D. Nagin, A. Chouldechova, On the validity of arrest as a proxy for offense: Race and the likelihood of arrest for violent crimes, in *Proceedings of the 2021 AAAI/ACM Conference on AI, Ethics, and Society* (ACM, 2021), pp. 100–111.

30. D. Knox, C. Lucas, W. K. T. Cho, Testing causal theories with learned proxies. *Annu. Rev. Polit. Sci.* **25**, 419–441 (2022).

31. J. Wang, Y. Liu, C. Levy, Fair classification with group-dependent label noise, in *Proceedings of the 2021 ACM conference on Fairness, Accountability, and Transparency* (ACM, 2021), pp. 526–536.

32. R. Fogliato, A. Chouldechova, M. G'Sell, Fairness evaluation in presence of biased noisy labels, in *2020 International Conference on Artificial Intelligence and Statistics* (ACM, 2022), pp. 2325–2336.

33. E. Pierson, D. M. Cutler, J. Leskovec, S. Mullainathan, Z. Obermeyer, An algorithmic approach to reducing unexplained pain disparities in underserved populations. *Nat. Med.* **27**, 136–140 (2021).

34. S. Mullainathan, Z. Obermeyer, On the inequity of predicting A while hoping for B, in *AEA Papers and Proceedings* (AEA, 2021), vol. 111, pp. 37–42.

35. J. Pearl, Linear models: A useful "microscope" for causal analysis. *J. Causal Infer.* **1**, 155–170 (2013).

36. K. Beckett, K. Nyrop, L. Pfingst, Race, drugs, and policing: Understanding disparities in drug delivery arrests. *Crim.* **44**, 105–137 (2006).

37. W. Cai, J. Gaebler, J. Kaashoek, L. Pinals, S. Madden, S. Goel, Measuring racial and ethnic disparities in traffic enforcement with large-scale telematics data. *PNAS Nexus* **1**, pgac144 (2022).

38. A. D. Biderman, A. J. Reiss Jr, On exploring the "dark figure" of crime. *Ann. Am. Acad. Pol. Soc. Sci.* **374**, 1–15 (1967).

39. Sewall Wright. Systems of mating. I. The biometric relations between parent and offspring. *Genetics* **6**, 111– 123 (1921).

40. M. L. Eaton, *Multivariate Statistics: A Vector Space Approach* (John Wiley & Sons Inc., 1983).
